# Biosynthesis of phyto-functionalized silver nanoparticles using olive fruit extract and evaluation of their antibacterial and antioxidant properties

**DOI:** 10.3389/fchem.2023.1202252

**Published:** 2023-05-30

**Authors:** Sami Ullah, Rimsha Khalid, Muhammad F. Rehman, Muhammad I. Irfan, Azhar Abbas, Ali Alhoshani, Farooq Anwar, Hatem M. A. Amin

**Affiliations:** ^1^ Institute of Chemistry, University of Sargodha, Sargodha, Punjab, Pakistan; ^2^ Department of Pharmacology and Toxicology, College of Pharmacy, King Saud University, Riyadh, Saudi Arabia; ^3^ Department of Food Science, Faculty of Food Science and Technology, Universiti Putra Malaysia, Serdang, Selangor, Malaysia; ^4^ Chemistry Department, Faculty of Science, Cairo University, Giza, Egypt; ^5^ Analytical Chemistry II, Faculty of Chemistry and Biochemistry, Ruhr University Bochum, Bochum, Germany

**Keywords:** silver nanoparticles, surface functionalization, characterization, antibacterial activities, disk diffusion, radical scavenging, *Olea europaea* L.

## Abstract

The green synthesis of nanomaterials is of utmost interest as it offers an eco-friendly approach over chemical synthetic routes. However, the reported biosynthesis methods are often time-consuming and require heating or mechanical stirring. The current study reports a facile one-pot biosynthesis of silver nanoparticles (AgNPs) mediated by olive fruit extract (OFE) and sunlight irradiation of only 20 s. OFE acts as both a reducing and a capping agent for the formation of OFE-capped AgNPs (AgNPs@OFE). The as-synthesized NPs were systematically characterized by UV-vis spectrometry, Fourier transform infrared (FTIR) spectroscopy, scanning electrochemical microscopy with energy-dispersive X-ray (SEM-EDX), X-ray diffraction (XRD), dynamic light scattering (DLS), and cyclic voltammetry. SEM images confirmed the successful formation of monodispersed spherical AgNPs@OFE of approximately 77 nm. FTIR spectroscopy suggested the involvement of functional groups of phytochemicals from the OFE in the capping and reduction of Ag^+^ to Ag. The particles revealed excellent colloidal stability as evidenced from the high zeta potential (ZP) value (−40 mV). Interestingly, using the disk diffusion method, AgNPs@OFE revealed higher inhibition efficiency against Gram-negative bacteria (*Escherichia coli*, *Klebsiella oxytoca*, and extensively drug-resistant (XDR) *Salmonella typhi*) than Gram-positive bacteria (*Staphylococcus aureus*), with *Escherichia coli* showing the highest inhibition zone of 27 mm. In addition, AgNPs@OFE exhibited maximum potent antioxidant scavenging potential against H_2_O_2_, followed by DPPH, O_2_^−^, and OH^−^ free radicals. Overall, OFE can be considered an effective source for the sustainable production of stable AgNPs with potential antioxidant and antibacterial activities for biomedical applications.

## 1 Introduction

The rapid emergence of antibiotic resistance in pathogenic bacteria embodies serious problems in hospitals and other healthcare units (Kuss et al., 2018). For example, pneumonia-like symptoms and urinary tract or wound infections are closely related to *Klebsiella oxytoca* (Herzog et al., 2014). Similarly, some *E. coli* strains can cause diarrhea and vomiting, and excessive exposure sometimes leads to kidney failure (Money et al., 2010). Specific strains of *S. aureus* have developed resistance to multiple antibiotics (Abbasi et al., 2021). The XDR *S. typhi* strain also shows resistance to fluoroquinolone or ceftriaxone, and its outbreak accounts for more than 20,000 cases every year (Rasheed et al., 2020). Hence, there is a prompt need to develop new antibiotics to cope with the challenge of multiple-drug-resistant bacteria.

Nanoparticles have attracted tremendous attention in the last decades due to their unique optical, chemical, magnetic, biological, and electrical properties that differ from those exhibited by their respective bulk materials (Atta et al., 2012; Tschulik et al., 2014; Amin et al., 2018; dos Santos et al., 2019; Abbas and Amin, 2022). Among others, silver nanoparticles (AgNPs) are being intensively utilized in a variety of applications, including chemical and electrochemical sensing (Amin et al., 2022), catalysis (Amin et al., 2015; Amin et al., 2017), nanomedicine (Lee and Jun, 2019), agriculture (Gao et al., 2022), cosmetics (Fukui, 2018), and antioxidants (Sharma et al., 2012). Furthermore, AgNPs demonstrated antiviral (Jeremiah et al., 2020), antibacterial (Ocsoy et al., 2013; El Arrassi et al., 2017; Amin et al., 2022), and antifungal potential (Huang et al., 2020). Consequently, AgNPs have been widely used as effective antibacterial agents in water treatment, antiseptic sprays, wound dressings, topical creams, and fabrics (Hebeish et al., 2014). In this context, the facile and sustainable fabrication of stable AgNPs with remarkable antibacterial performance is indispensable. In a recent review, the advances in the preparation and characterization of AgNP-based electrodes and their physicochemical properties were comprehensively reported (Abbas and Amin, 2022).

Although conventional chemical and physical synthesis methods may produce pure and well-defined NPs, they are energy-intensive, expensive, and potentially hazardous to the environment (Machado et al., 2019). As a green alternative, plant extract-mediated synthesis of AgNPs has gained significant attention in recent years due to its potential as an eco-friendly and cost-effective method for producing nanoparticles with antibacterial properties (Khan and Javed, 2021). Various plant species, such as *Azadirachta indica* (Khan and Javed, 2021), raspberries and blackberries (*Rubus)* (Ekrikaya et al., 2021), *Abelmoschus esculentus* (Devanesan and AlSalhi, 2021), and *Piper colubrinum* (Santhoshkumar et al., 2021), have been reported for the biosynthesis of AgNPs, making it a versatile approach. Olive (*Olea europaea* L.) is recognized as a substantial source of natural antioxidants (Rodríguez De Luna et al., 2020), having potential in various biological activities such as, antiaging (Castejón et al., 2020) and antimicrobial effects (Karygianni et al., 2019). The main bioactive compounds present in olive fruit (OF) are phytochemicals including flavonoids, phenolic acids, terpenoids, phenolic alcohols, sugars, proteins, alkaloids, secoiridoids, and hydroxycinnamic acid derivatives (Ghanbari et al., 2012), which can act as reducing agents, stabilizers, and capping agents during nanoparticle synthesis. Natural antioxidants extracted from plants, especially phenolics and flavonoids (*e.g*., ferulic acid, gallic acid, kaempferol, quercetin, and catechins), have been examined as antioxidant-based antibacterial agents and demonstrated promising effects to overcome the resistance of various microbes versus synthetic antibiotics (Rajesh et al., 2020). Additionally, when combined with silver nanoparticles, plant extracts can exhibit synergistic effects, enhancing their antibacterial activity and colloidal stability compared to other traditional capping agents and biomolecules (Some et al., 2020).

Although the green synthesis of nanoparticles using plant extracts offers several advantages, it is also associated with some challenges that should be considered: achieving consistency and uniformity in the nanoparticles’ properties such as size, shape, or biological activity can be difficult due to the natural variations in plant extracts in terms of composition and bioactive compounds (Ceylan et al., 2021). For instance, AgNPs were synthesized using extracts of three *Sideritis* species, and the resulting NPs revealed different bioactivities, although having similar particle size (22–26 nm). Indeed, plant extracts have been widely used for the synthesis of functionalized NPs; however, a major drawback of these studies is often the lack of information about the main compounds in the extracts used. Thus, chemical analysis for the identification of the extract constituents is important. In addition, few further shortcomings of some reported biosynthesis procedures include the use of heat for the reduction, the relatively long time of synthesis, or the limited colloidal stability of the fabricated nanoparticles (Ekrikaya et al., 2021). Thereby, developing sustainable, cost-, and energy-effective routes for the synthesis of stable AgNPs within a short time is still important.

In the present study, to the best of our knowledge, we demonstrate for the first time the use of olive fruit extract in the green synthesis of AgNPs via sunlight irradiation for only tens of seconds. The morphology, composition, size, and colloidal stability of the as-synthesized particles (AgNPs@OFE) were systematically characterized. The combination of AgNPs with an OFE capping agent exhibited synergistic antibacterial activity. In addition, we verify the application of these particles as antioxidant scavenging agents for DPPH, H_2_O_2_, O_2_^−^, and OH^−^radicals.

## 2 Materials and methods

### 2.1 Materials

Olive fruits (OFs) were identified and manually collected from 10 Koroneiki olive trees (coded as KFORT-1 to KFORT-10) by the Barani Agriculture Research Institute (BARI), Chakwal, Punjab, Pakistan, during October 2020. Olive plants aged 8–10 years, and the used fruits were ripe with an average weight of 3 g and size of 2–3 cm. Analytical grade silver nitrate (99.98%) was purchased from Merck, Germany. All solutions were prepared using distilled water. The bacterial strains of *S. typhi*, *S. aureus*, *K. oxytoca*, and *E. coli* were kindly provided by Department of Biochemistry (Institute of Chemistry, University of Sargodha, Punjab, Pakistan).

### 2.2 Preparation of olive fruit extract (OFE)

**SCHEME 1:**
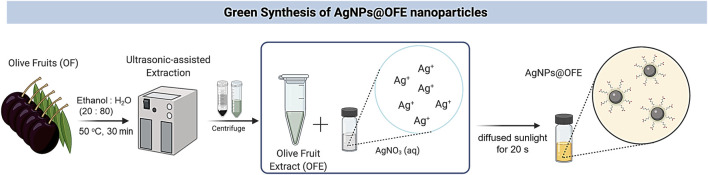
Schematic illustration for the biosynthesis of AgNPs using olive fruit extract and sunlight irradiation.

Healthy fruits without any physical damage or infection (featured by reddish-brown spots or fungal spores) were selected. Fresh OF pulps (10 g) were utilized for ultrasound-assisted extraction with ethanol: water (80: 20 mL) solvent at 50°C for 30 min, as shown in Scheme 1. The sonicated solution was then filtered using nylon syringe filters (pore size of 0.45 µm), and the obtained extract was further defatted by n-hexane partitioning. Afterward, the extract was dried in a water bath at 45°C to obtain a semi-solid form.

### 2.3 GC-MS analysis of the OFE

To identify the extract constituents, GC-MS analysis was conducted. The analysis was performed by gas chromatography (GC) (Agilent 5977A) coupled with MS (7890A, Agilent, United States), where 1 mL of the sample was injected into a DB-5MS fused-silica capillary column of 30 m length, 0.25 mm inner diameter, and 0.25 µm film thickness (J&W Scientific, Folsom, CA). Helium (99.9% pure) was used as the carrier gas (with a flow rate of 1 mL/min). The temperature of the column was maintained at 60°C for 2 min, followed by an increase to 250°C at a rate of 5°C/min and kept constant at 250°C for 5 min. The mass spectrum was scanned (at a rate of 1.5 scans/s) from *m/z* 50 to 650. Peaks were identified using the MassHunter library, and the peak area (%) was used to determine the relative percentage of compounds present in the tested sample.

### 2.4 Synthesis of OFE-functionalized AgNPs

AgNPs@OFE were synthesized by mixing 25 mL of diluted solution of OFE (100 μg/mL) and 25 mL of AgNO_3_ (100 mM) in a glass vial, which was then directly irradiated under diffused sunlight outdoors at various exposure times, as shown in Scheme 1. We note that the borosilicate glass container used for the synthesis filters out part of the UV light, and thus, mainly IR and visible lights were incident onto the solution. The color of the mixture changed from colorless to light yellow almost instantly, indicating that AgNPs have been successfully synthesized. It should be noted that without exposure of the mixture solution to sunlight, no color change was observed, indicating its essential role in the synthesis. The AgNPs were then centrifuged at 13,000 rpm for 30 min. The supernatant was subsequently separated from the AgNPs by the decantation method, where the top liquid layer in the tube was poured out, leaving the precipitated solid part at the bottom. Then, distilled water (25 mL) was added to the nanoparticles and centrifuged again for 10 min. The supernatant was again separated by decantation. This washing step of AgNPs was repeated thrice to remove any unreacted extract or AgNO_3_. The AgNPs were then re-dispersed in water (5 mL) by sonication in an ultrasonic bath (35 kHz) at 25°C for 15 min. The resulting suspension of AgNPs was used as the stock solution for further spectroscopic characterizations and applications. A UV-vis spectrophotometer was used to examine the progress of AgNPs formation over various reaction times.

### 2.5 Determination of the concentration of synthesized AgNPs@OFE

The number of silver atoms in one silver nanoparticle and the concentration of the suspension of the synthesized silver nanoparticles (AgNPs@OFE) were obtained as follows (Ngamchuea et al., 2017):

$$The\;diameter\;\left(d\right)\;of\;the\;particle\;\left(obtained\;from\;SEM\right)\;is:d=77.1\;nm,$$ (1)

$$Thus,the\;radius\;\left(r\right)\;equals:r=38.6\times 10^{-9}m,$$ (2)

$$\begin{array}{c} The\;volume\;\left(V\right)\;of\;a\;particle:V=\frac{4}{3}\Pi \;r^{3}=\frac{4}{3}\times 3.\;14\times {\left(38.6\times 10^{-9}\right)}^{3}\;m^{3},\\ V=2.41\times 10^{-22}\;m^{3}. \end{array}$$ (3)

Using Avogadro’s number $N_{A}=6.02\times 10^{23}$ , the density of Ag $\rho =10.49\;g\;cm^{-3}$ , and the atomic mass of Ag $M=107.\;87\;g\;{mol}^{-1}$ , the number of silver atoms (*N*) in one nanoparticle was calculated as follows:

$$N=\left(V*N_{A}*\rho \right)/M={1.4\times 10}^{7}Particles.$$ (4)

Furthermore, the molar absorptivity $\left(Ɛ\right)$ for AgNPs@OFE can be calculated according to the following equation:

ln⁡ε=1.4418ln⁡d+18.955, (5)

where *d* is the diameter of the AgNPs in nm. The ε value was calculated using Eq. 5 for a particle of size 77.1 nm (from SEM) and was found to be 9.3 × 10^10^ M^-1^ cm^-1^. Using the parameters from the UV-vis measurement of absorbance *A* = 1.8 and path length l=1 cm , the concentration of the AgNPs@OFE was calculated using the Beer–Lambert law as follows:

$$c=\frac{A}{\varepsilon \times l}=\frac{1.8}{9.3\times 10^{10}\times 1}=1.1\times 10^{-10}M=0.02\;nM$$ (6)

### 2.6 Characterization of AgNPs@OFE

AgNPs and OFE were characterized using a UV-vis double-beam spectrophotometer (UV-1800 spectrophotometer, Shimadzu, Japan), which consisted of a deuterium and tungsten lamp with a wavelength range of 200–800 nm at room temperature. The Fourier transform infrared (FTIR) spectra (in the range of 400–4,000 cm^−1^) of both OFE and AgNPs@OFE were recorded using an FTIR spectrometer (Shimadzu, Japan). The size and surface morphology of the synthesized particles were examined using a scanning electron microscope (SEM) (Nova NanoSEM NPE 218), which was coupled with an energy-dispersive X-ray (EDX) unit (JEOL EDX system) for elemental analysis. The AgNPs@OFE sample was dispersed in water and was then drop-cast on a glassy carbon plate for analysis. The AgNPs@OFE sample was also characterized by X-ray diffraction (XRD) to determine the crystalline structure and size of the crystallites (Philips X'Pert-PRO X-ray diffractometer system). The X-ray tube was operated at a voltage of 40 kV, while the beam current was 30 mA. The 2θ range of the XRD patterns was between 10° and 90° under CuK_α_ = 0.154 nm. Scan speed during the analysis was maintained at 5°/min.

Additionally, the particle size distribution in the solution was measured by dynamic light scattering (DLS) (Nano ZS Zetasizer system, Malvern Instruments) operating with a He–Ne laser (633 nm) and at 25°C. A medium (water) refractive index of 1.33, Ag refractive index of 0.20, and a medium viscosity of 0.89 mPa⋅s were used. Prior to the DLS measurements, the particle suspension was filtered using a polyvinylidene fluoride (PVdF) membrane of pore size 0.2 *μ*m. The sample was then loaded into a clean quartz micro-cuvette, and five repeated measurements were performed and the average size was calculated. Furthermore, the electrochemical characterization was conducted using a three-electrode arrangement on a GAMRY potentiostat interface 1010E (Unites States). The working electrode was a glassy carbon electrode (GCE, 3 mm) modified with the AgNPs by drop-casting (0.5 µL). A saturated calomel electrode (SCE) and platinum wire were used as the reference and counter electrodes, respectively. Prior to drop-casting of the particles, the electrode was polished to a mirror finish with alumina slurry in the sequence of sizes 3.0, 1.0, and 0.1 *μ*m on a polishing pad. Cyclic voltammograms (CVs) were recorded for AgNPs@OFE on the GCE in the potential window of −1.0 to +1.0 V at a scan rate of 100 mV s^-1^ in 0.1 M KCl solution.

### 2.7 Antibacterial activity of AgNPs@OFE

The antibacterial activity of OFE and AgNPs@OFE was tested against four species of bacterial pathogens, namely, *S. aureus*, XDR *S. typhi*, *E. coli*, and *Klebsiella oxytoca*, using the disk diffusion method. Nutrient agar media were prepared by mixing agar (16 g), sodium chloride (8 g), tryptone (8 g), and yeast (4 g) in distilled water to obtain a volume of up to 400 mL in a flask. pH was maintained at 7.4 before medium sterilization (Aslam et al., 2011). The bacteria were spread on Petri plates, and then disks with four different concentrations of OFE or AgNPs@OFE were placed over the Petri plates. These antibacterial disks were made of Whatman filter papers of diameter 6 mm (Grade 41, pore size of 20–25 μm, and 220 µm thickness). The plates were then incubated for 24 h at 37°C. The tested bacterial suspension was 10^7^ CFU mL^−1^. The diameter (in mm) of the zone of inhibition (ZoI) was measured to assess the antimicrobial activity. The assay was repeated thrice, and the values were noted as the mean ± standard deviation. Both OFE and AgNPs@OFE samples were studied for the final concentrations of 10, 20, 30, and 40 μg mL^−1^ aqueous solution, which were prepared by taking 10, 20, 30, and 40 µL from 100 μg mL^−1^ stock solution, respectively.

### 2.8 Antioxidant assays

#### 2.8.1 Hydrogen peroxide (H_2_O_2_) scavenging activity

The used procedure followed a previously reported method (Aslam et al., 2011). Briefly, 100 µL of 50 μg mL^−1^ AgNPs@OFE was dissolved in 50 mM phosphate-buffered solution (pH 7.4), and then, 600 µL of 2 mM H_2_O_2_ solution was added to it. The resulting mixture was vortexed, and after 10 min, an absorbance at 230 nm was measured using a UV-vis spectrophotometer. Ascorbic acid in the phosphate buffer was also used as the reference material. The scavenging percentage of hydrogen peroxide and other studied radicals was calculated according to Eq. 7.

$$Scavenging\;\left(\%\right)=\frac{F_{c}-F_{s}}{F_{c}}\times 100,$$ (7)

where *F*_*c*_ and *F*_*s*_ represent the absorbance for the control (ascorbic acid in buffer) and sample AgNPs@OFE + buffer solutions, respectively.

#### 2.8.2 2, 2-Diphenyl-1-picrylhydrazyl (DPPH) activity

The method was adapted from a procedure proposed by Bhakya et al. (2016) with slight modifications. Briefly, 1 mL of (50 μg mL^−1^) AgNPs@OFE was mixed with 1 mL of freshly prepared DPPH (1 mM in methanol) and was then shaken thoroughly. The mixture was incubated at 30°C in the dark for 30 min. The absorbance at 517 nm was recorded. The control solution was 1 mL DPPH plus 1 mL of methanol, while pure methanol was used as the blank.

#### 2.8.3 Hydroxyl radical (HO^−^) scavenging activity

The applied method follows the protocol reported by Keshari et al. (2016). Here, 600 μL of AgNPs@OFE + ascorbic acid (50 μg mL^−1^ in methanol), 120 µL of 10 mM FeSO_4_–EDTA, 360 µL of 200 mM sodium phosphate buffer (pH 7.0), 120 µL of 10 mM deoxyribose, 120 µL of 10 mM H_2_O_2_, and 420 µL deionized water were thoroughly homogenized. After incubation (for 4 h), the reaction of the resulting mixture was ceased by adding 600 µL of 2.8% trichloroacetic acid and 600 µL of 1% TBA (in 50 mM NaOH). After that, the solution was placed in a boiling water bath for 10 min and then cooled with tap water. The absorbance at 520 nm was recorded. Methanol was used as a blank.

#### 2.8.4 Superoxide radical (O_2_^−^) scavenging activity

This scavenging activity was assessed as described by Keshari et al. (2016). Superoxide radicals were produced in the phenazine methosulfate–nicotinamide adenine dinucleotide (PMS-NADH) system by the oxidation of NADH, and the activity was measured by monitoring the decrease in the reduction of nitro blue tetrazolium (NBT). Briefly, 500 µL of 16 mM Tris-HCl buffer (pH = 8), 500 µL 10 mM PMS, 500 µL of 78 mM NADH, 100 µL of AgNPs@OFE (50 μg mL^−1^), and 500 µL of 50 mM NBT were thoroughly mixed and maintained for 5 min at 27°C. The absorbance at 560 nm was recorded.

In all antioxidant activities, ascorbic acid was used as the standard material.

## 3 Results and discussion

The successful biosynthesis and characterization of AgNPs@OFE and evaluation of their antibacterial and antioxidant activities are discussed in this section. The AgNPs@OFE were characterized using diverse modern techniques such as UV-vis, FTIR, SEM, EDX, DLS, ZP, and cyclic voltammetry.

### 3.1 GC-MS analysis of the OFE

**FIGURE 1:**
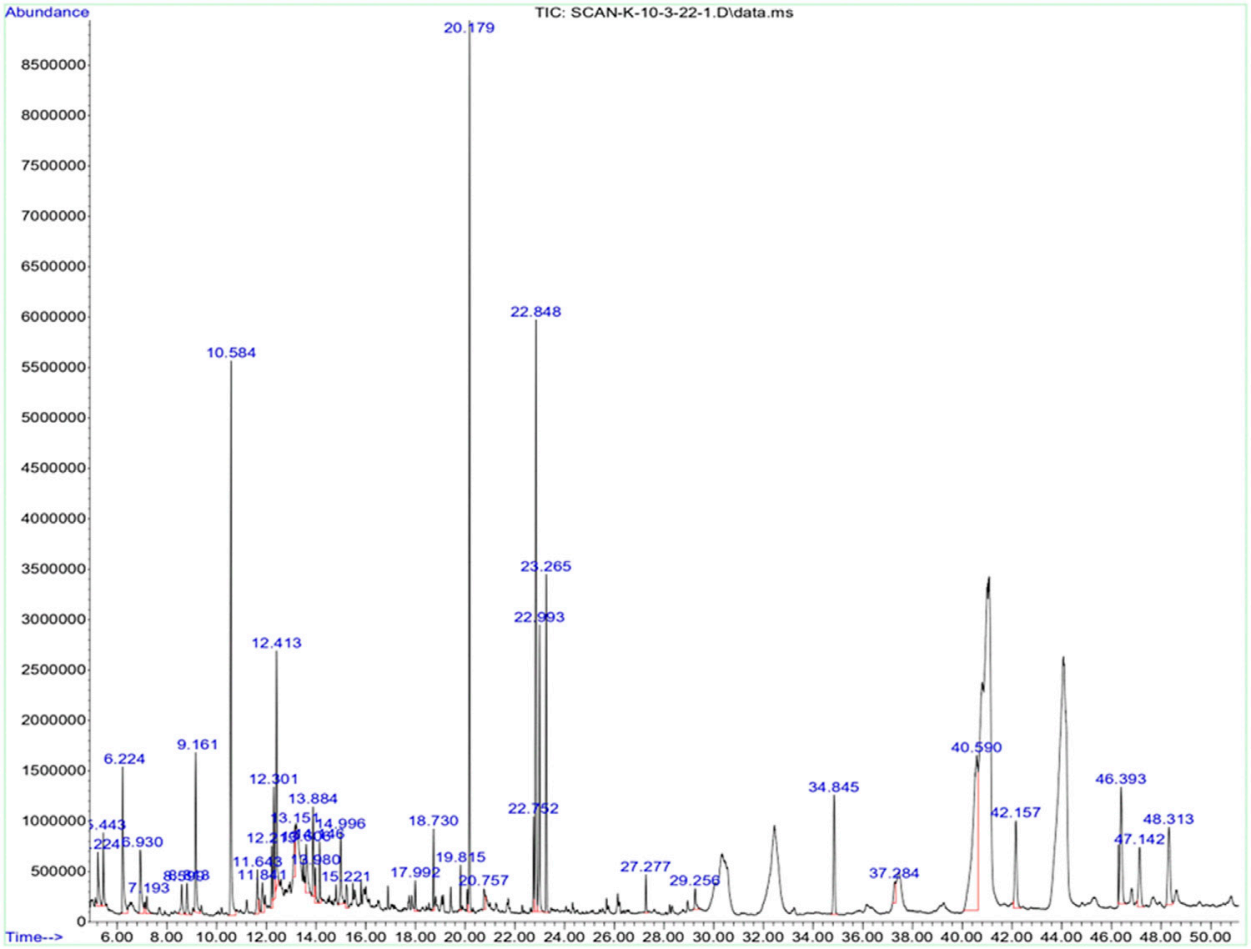
GC-MS chromatogram of olive fruit extract.

To elucidate the major bioactive compounds of the extract that are responsible for the reduction and capping of the synthesized AgNPs, GC-MS analysis was performed. Chromatographic methods could provide more precise information on the capping agent than FTIR, in which the detected functional groups and bonds could occur across many phytochemicals. Figure 1 displays the chromatogram of the OFE. A total of 35 compounds were identified, and their chemical structure, retention time, and concentration (peak area %) are given in Supplementary Figure S1 and Supplementary Table S1. As expected, the extract is rich in polyphenols, triterpenes, and fatty acids. The identified phenolic compounds, vitamin E (*α*-tocopherol), fatty acids, and triterpenes such as *α*-amyrin, *β*-amyrin, and *α-* and *β*-sitosterol have strong antioxidant potential. Furthermore, these phytochemicals (phthalic acid, sinapic acid, benzoic acid and its derivatives, tyrosol, homovanillyl alcohol, and orcinol) have also been reported as antimicrobial agents.

### 3.2 Biosynthesis and physicochemical characterization of AgNPs@OFE

#### 3.2.1 UV-vis characterization

**FIGURE 2:**
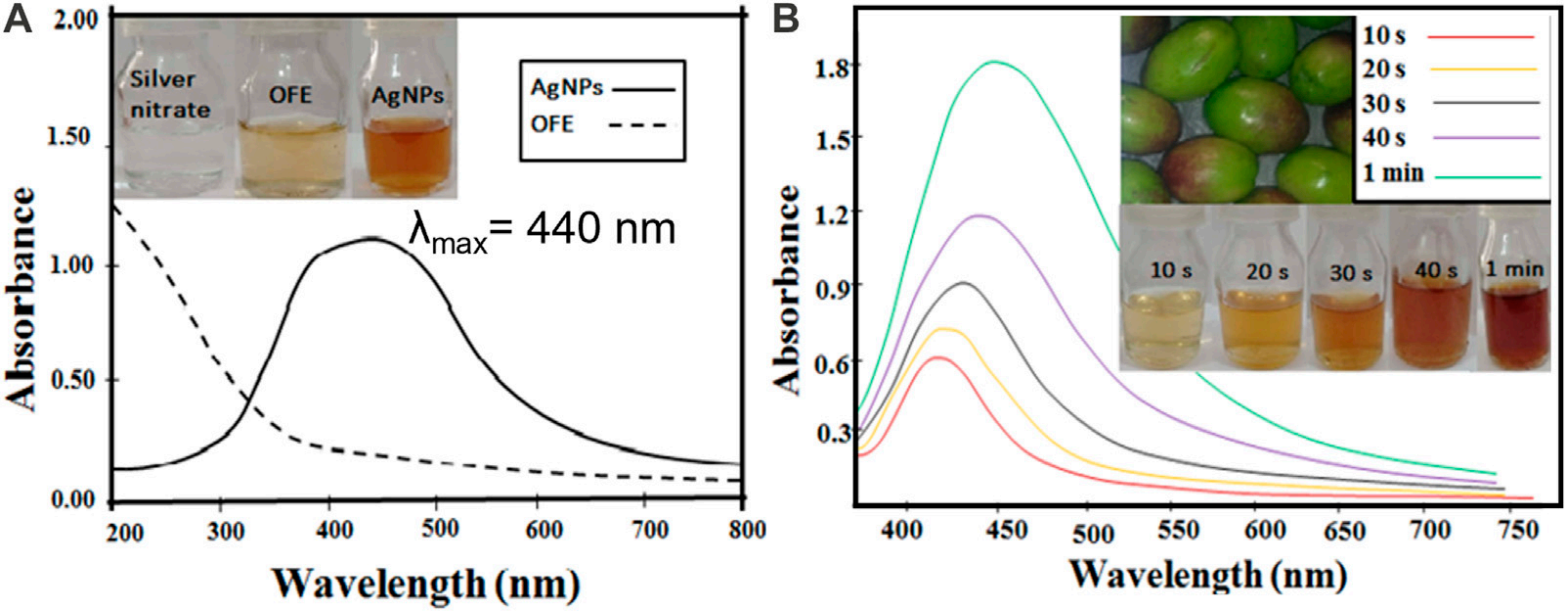
**(A)** UV-vis spectra of the as-synthesized AgNPs@OFE and OFE. **(B)** UV-vis spectra of the AgNPs@OFE synthesized at different exposure times to sunlight. The insets show the colorimetric responses of the respective solutions.

The OFE-capped AgNPs were synthesized by mixing a dilute solution of OFE and AgNO_3_, followed by irradiation under diffused sunlight at various exposure times. Although the source of light and temperature might affect the synthesis of nanoparticles, in this study, we focus on optimizing one procedure and evaluating the properties of the synthesized particles rather than comparing different synthetic routes with many parameters. The color of the reaction mixture changed from colorless to light yellow and then to brownish-yellow after exposure to sunlight (see the inset of Figure 2A), indicating the formation of plasmons at the colloidal surface and thus the synthesis of AgNPs@OFE. The color change is due to the reduction of Ag^+^ ions to metallic Ag by the reducing agents in the phytochemicals in the extract and the sunlight. The UV-vis spectrum given in Figure 2A clearly shows a single localized surface plasmon resonance (LSPR) peak centered at 440 nm, proving the successful synthesis of spherical AgNPs@OFE, while the OFE solution shows no absorption band (Rycenga et al., 2011). This agrees with our previous work on the sunlight-mediated synthesis of sulfonamide-functionalized AgNPs (Amin et al., 2022). The effect of sunlight exposure on the synthesis of AgNPs@OFE was examined by UV-vis spectrometry at a 10 s exposure time interval, as shown in Figure 2B. A fresh solution from the OFE + Ag^+^ stock was used for the successive irradiations to avoid time delay between the irradiation and UV-absorption measurements. The results show that as the exposure time increases, the color of AgNPs@OFE dispersion intensifies with higher absorbance intensity, and a redshift in the LSPR position occurs. This increase in absorbance is due to a progressive conversion of silver ions into AgNPs, consequently increasing their concentration in the medium. In addition, the gradual broadening of the UV-vis spectra, together with the redshift of their maxima (λ_max_), is due to a decrease in interparticle spacing and further nucleation and growth of AgNPs@OFE forming relatively larger particles (Abbas et al., 2020). This observation indicates that sunlight irradiation is effective and crucial in the successive growth of AgNPs@OFE. Additionally, the UV-vis spectra revealed that the formation of AgNPs@OFE occurred rapidly within 20 s, indicating that the OFE hastens the biosynthesis of silver nanoparticles. Accordingly, we suggest that 20 s is an optimal time for the formation of monodispersed AgNPs@OFE, whilst at longer durations, agglomerates are prone to form. With respect to the very short synthesis time (20 s), sustainability (no hazardous reducing agents), and low cost (no sophisticated setups are required), our synthetic route is compared favorably to traditional chemical reduction or vapor deposition methods.

The mechanism of formation of AgNPs using OFE involves a series of reduction, nucleation, and stabilization steps, as illustrated in Scheme 1 (Mikhailova, 2020). i) Reduction step: This step involves the transfer of electrons from the bioactive compounds to silver ions, resulting in their reduction to elemental silver. ii) Nucleation: The reduced silver ions undergo nucleation, where small clusters of silver atoms are formed. These clusters act as the initial building blocks for the growth of silver nanoparticles. The presence of phytochemicals in the plant extract facilitates the nucleation process and promotes the formation of stable nuclei. iii) Growth: This step involves the aggregation and growth of the silver atoms into larger nanoparticles, with the phytochemicals in the OFE controlling the growth process and influencing the size, shape, and morphology of the nanoparticles. The stabilizing role of the phytochemicals helps prevent the agglomeration of the nanoparticles and maintain their individuality in the solution.

#### 3.2.2 FTIR analysis

**FIGURE 3:**
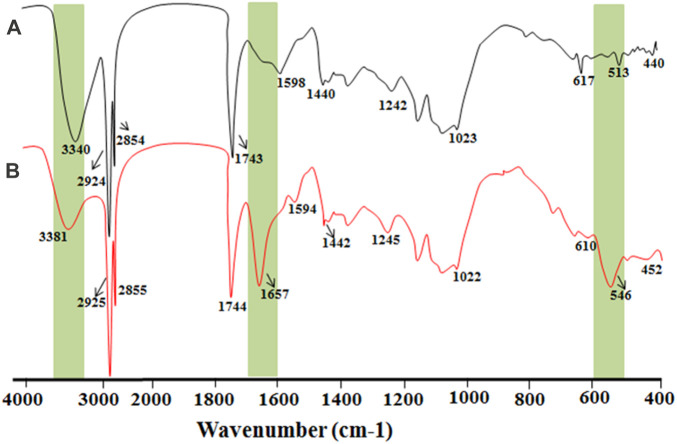
Overlay of the FTIR spectra of **(A)** pure OFE and **(B)** AgNPs@OFE.

To confirm the reduction and capping of AgNPs with the OFE, the FTIR spectra of both OFE and AgNPs@OFE were recorded, as depicted in Figure 3. The probable functionalities and biomolecules in the OFE were identified by the FTIR analysis. The absorption spectra arise from the chemical groups in contact with AgNPs@OFE since the samples were centrifuged and washed thrice to remove non-adsorbed chemical groups. The spectral data suggest that the OFE contains flavonoids, alkaloids, steroids, phenolic acids, carboxylic acids, secoiridoids, and proteins, according to previous phytochemical examinations (Rajashekar et al., 2012).

Importantly, comparing the peaks of the spectra of OFE and AgNPs@OFE revealed the retention of all peaks of OFE in the FTIR spectrum of AgNPs@OFE with only slight shifts in peak positions. In the case of sole OFE, FTIR peaks were observed at 440, 513, 617, 1,023, 1,242, 1,450, 1,598, 1,743, 2,854, 2,924, and 3,340 cm^−1^, and for the AgNPs@OFE, the respective bands were observed at 452, 546, 610, 1,022, 1,245, 1,442, 1,594, 1,657, 1,744, 2,855, 2,925, and 3,440 cm^−1^. The assignment of these peaks to the respective functional groups and compounds is discussed as follows: this retention of the peaks in the FTIR spectrum of AgNPs@OFE proves the involvement of functional groups of OFE compounds in the reduction of silver ions to AgNPs and the functionalization of the resulting particles. On the other hand, few new FTIR peaks emerged/intensified for the AgNPs@OFE. For example, a band at approximately 546 cm^−1^ appeared for the AgNPs@OFE sample, which could be assigned to the bending vibrations of O–Ag. The peak at 440 cm^−1^ of the OFE can be attributed to a ring-opening vibration and was shifted toward a higher wavenumber (452 cm^−1^) in the AgNP spectrum. The band observed at 617 cm^−1^ in the OFE spectrum was displaced to 610 cm^−1^ in AgNPs@OFE and corresponded to C–Cl stretching in an alkyl group. We suggest that the negatively charged AgNPs (as evidenced from the negative zeta potential value) could attract the positively charged chemical groups from the extract, which most probably led to the changes observed in the FTIR spectra. The C–N stretch vibrations, as well as the amide bands of proteins in the fruit extract, were reflected in the intense peaks at 1,242 and 1,245 cm^−1^ for the fruit extract and AgNPs, respectively (Gurunathan et al., 2015). Furthermore, the peak at 1,022 cm^-1^ in the AgNPs@OFE spectrum was assigned to ether linkages and suggested the presence of flavanones adsorbed on the AgNPs@OFE (Shankar et al., 2004). The peak at 1,654 cm^−1^ could mostly be assigned to amide I, stretching vibrations of the aromatic ring, or stretching vibrations of C=O groups. The peaks for the carbonyl of carboxylic acid groups were also present at approximately 1,744 cm^−1^ in the AgNPs@OFE spectrum (Jeeva et al., 2014). The C=C stretching in the aromatic ring was observed at 1,598 and 1,594 cm^−1^ in both spectra, confirming the presence of the aromatic group (Reddy et al., 2014). The O–H groups (at 3,340 cm^−1^) of polyphenols and carboxylic acids of OFE were also found to be present in AgNPs@OFE at 3,381 cm^−1^. This detailed analysis of both spectra demonstrates the role of flavonoids, phenolic acids, carboxylic acids, and proteins in the rapid reduction and capping of Ag^+^ to AgNPs@OFE (Zuas et al., 2014).

#### 3.2.3 Morphological, structural, and compositional characterization

**FIGURE 4:**
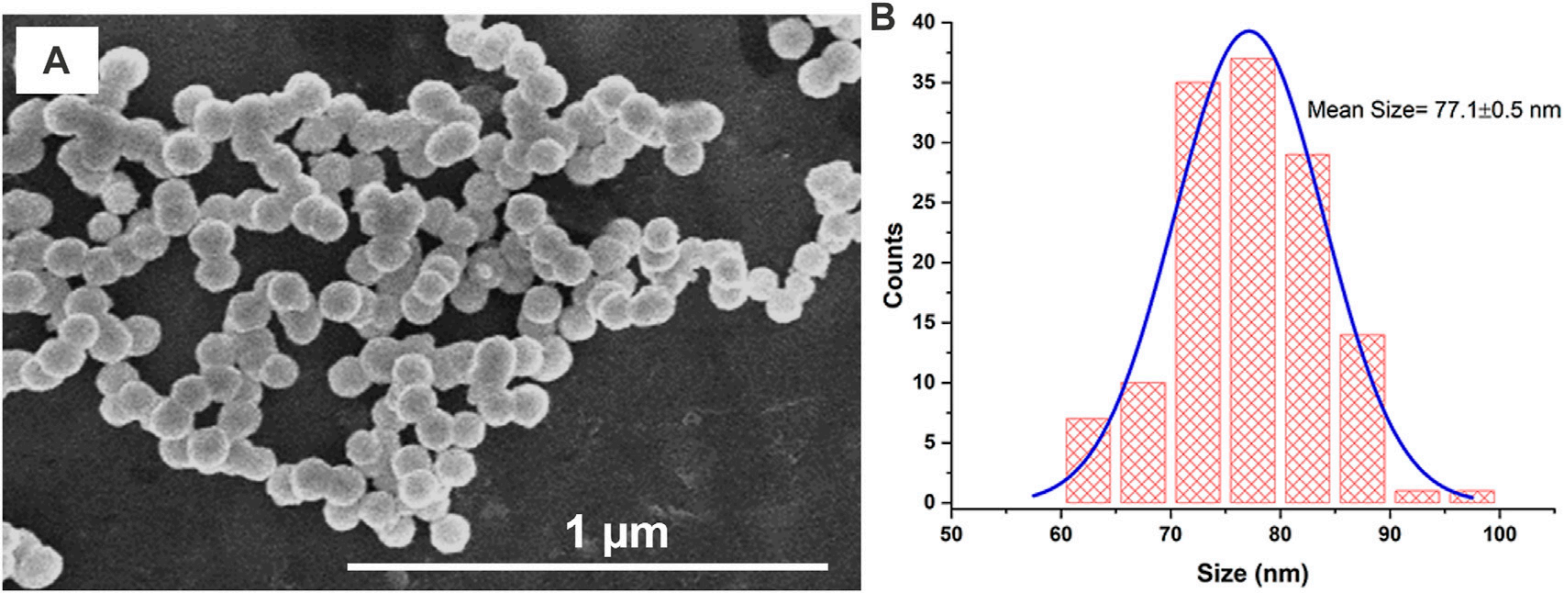
**(A)** SEM image of AgNPs@OFE and **(B)** histogram for the size distribution of AgNPs@OFE obtained from the respective SEM image in **(A)**.

SEM images were recorded to examine the particle morphology and size. Figure 4A displays an SEM image of the AgNPs@OFE sample synthesized at an exposure time of 25 s. The SEM image shows uniform spherical particles of AgNPs@OFE. The particle size was determined by SEM image analysis using ImageJ software, and the resulting sizes were used to establish a histogram for the size distribution, as shown in Figure 4B. The calculated average diameter was found to be 77.1 ± 0.5 nm. As an advantage of this synthesis method, the histogram shows a narrow particle size distribution.

**FIGURE 5:**
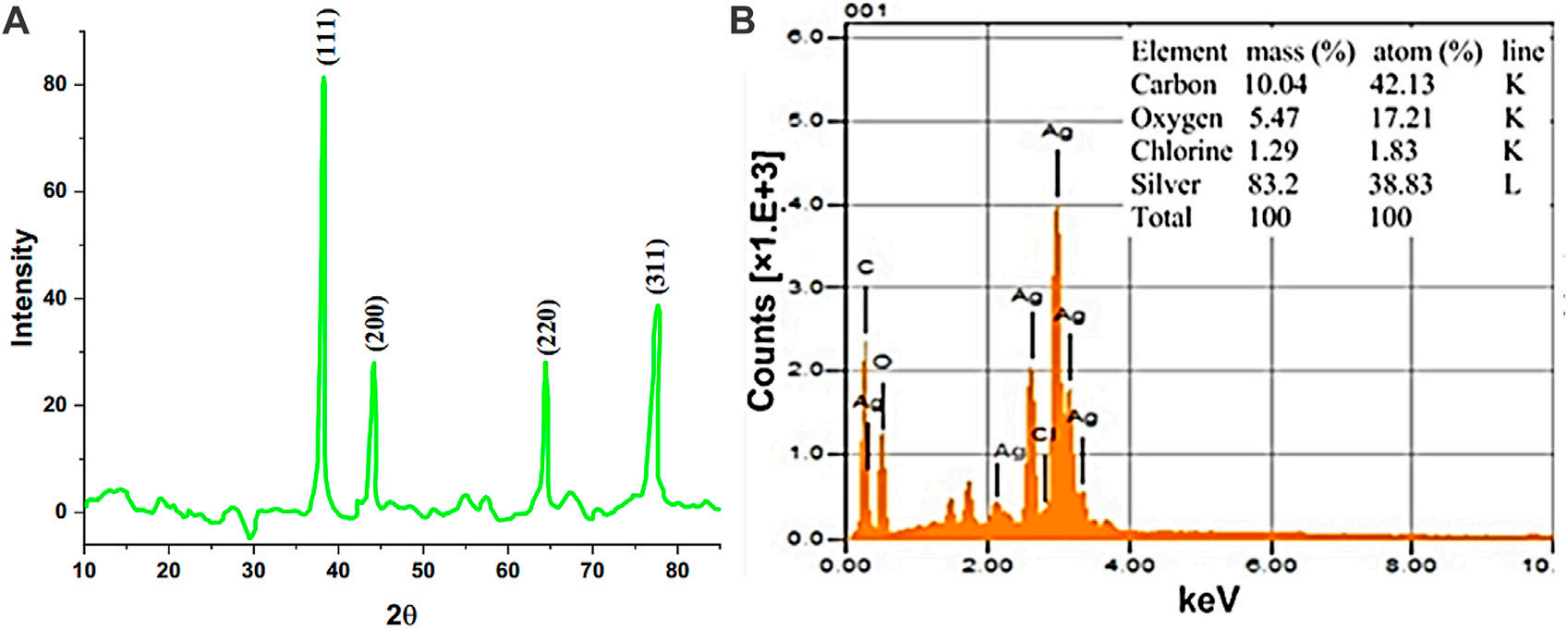
**(A)** XRD pattern of AgNPs@OFE and **(B)** EDX spectrum of AgNPs@OFE.

XRD analysis of AgNPs@OFE was carried out to confirm the crystalline nature of the particles synthesized after 25 s of irradiation. The XRD pattern of AgNPs@OFE exhibited characteristics of the Bragg peaks of silver nanocrystals, as shown in Figure 5A. The peaks observed at 2θ values of 38.16, 44.02, 64.30, and 77.59^o^ were linked to different diffraction lattice planes of (111), (200), (220), and (311), respectively, which is in agreement with previous reports (Amin et al., 2022). These set of lattice planes confirmed the face-centered cubic structure of AgNPs@OFE (JCPDS, File No. 04–0783) (Lanje et al., 2010). Some other sharp peaks were observed in addition to Ag peaks, which might be attributed to the presence of adipic acid capping around the AgNPs as the AgNPs used for the analysis were crude nanoparticles.

The Debye–Scherrer equation (Eq. 8) was used to assess the average particle size (i.e., crystallite size) of the AgNPs@OFE as follows:

$$D=\frac{K\lambda }{\beta \mathrm{Cos}\theta }$$ (8)

where D is the average nanoparticle size, *K* is the Scherrer constant with a value of 0.94 for spherical crystallite size with cubic symmetry, and *λ* is the wavelength of the X-ray used for diffraction (for CuK_α_ = 0.154 nm). “*β*” is the full width at half-maximum (FWHM), and “*θ*” is the diffraction angle. The values of *β*, i.e., FWHM in radian, were calculated for the main peaks in the XRD pattern shown in Figure 5B and were found to be 0.00215, 0.00196, 0.00209, and 0.00231 at 2θ values of 38.16, 44.02, 64.30, and 77.59, respectively. The average crystallite size using the Debye–Scherrer equation was calculated to be 78.41 nm, which is in close agreement with the size estimated from SEM.

EDX analysis was also performed to confirm the elemental composition, as shown in Figure 5B. Clear peaks of the Ag element were observed, proving the formation of AgNPs. The mass% of carbon, oxygen, chloride, and silver was 10.0, 5.5, 1.3, and 83.2, respectively, as given in the inset of Figure 5B. The C, O, and Cl possibly belong to chemical components of the bioactive compounds (constituents of OFE) that are responsible for capping and stabilizing the AgNPs.

**FIGURE 6:**
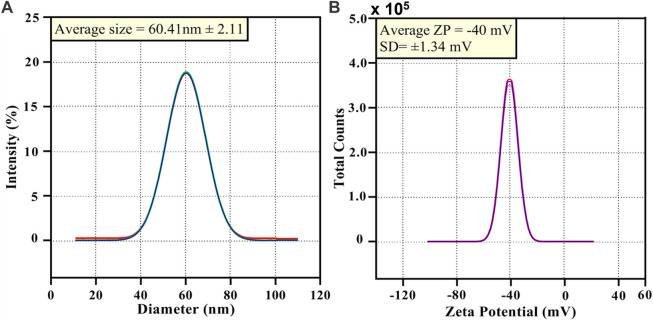
**(A)** DLS of AgNPs@OFE and **(B)** zeta potential data of AgNPs@OFE.

The size of the synthesized AgNPs@OFE (25 s of irradiation) was also obtained from DLS measurements, as shown in Figure 6A. The size distribution of the particles exhibited a narrow range (50–100 nm). The average hydrodynamic diameter of AgNPs@OFE in solution was found to be 60.4 **±** 2.1 nm. We note that the DLS size of the AgNPs@OFE is unexpectedly smaller than that determined from SEM images (77.1 ± 0.5 nm). This might be attributed to the fact that the SEM image represents only a very small fraction of the sample, while DLS probes a large quantity of particles or possible shrinking of the core diameter upon formation of a permeable polymer layer in contact with water in the DLS measurements (Fissan et al., 2014).

Furthermore, the zeta potential (ZP) was determined to identify the nature and magnitude of the surface charge of particles and to consequently evaluate the colloidal stability or aggregation in the solution. Accordingly, to assess the colloidal stability of AgNPs@OFE, ZP measurements were conducted. The ZP of AgNPs@OFE was calculated as −40 ± 1.3 mV (Figure 6B). The NPs with a ZP range from ±25 to ±50 mV are considered to be very stable particles. Above and below this range, the particles are considerably unstable. It is known that ZP values ˃ ± 30 mV suggest highly stable colloids (Ardani et al., 2017). The obtained ZP of –40 mV indicates that the AgNPs@OFE can form a highly stable colloid system in water. We note that the AgNPs@OFE suspension was stored in a cool, dark place for 15 days, and it did not exhibit any obvious colorimetric changes due to the presence of effective OFE capping agent. This ZP value of −40 mV suggests that the surface of the nanoparticles is negatively charged, creating stronger repulsion between neighboring nanoparticles. Thus, the particles are electrostatically stabilized, which demonstrates the colloidal stability of the OFE-stabilized AgNPs. This ZP is more negative than the value reported for AgNPs capped with the organic molecule sulfonamide in a similar procedure, suggesting its better colloidal stability (Amin et al., 2022). The negative charge could be attributed to the carboxylate groups COO^−^ in the OFE capping agent. Moreover, the presence of polyphenols in the extract could impart stability to the NPs due to their steric effect (Sperling and Parak, 2010; Mohammadlou et al., 2016). Therefore, the OFE plays an effective role in stabilizing the AgNPs.

#### 3.2.4 Electrochemical response

**FIGURE 7:**
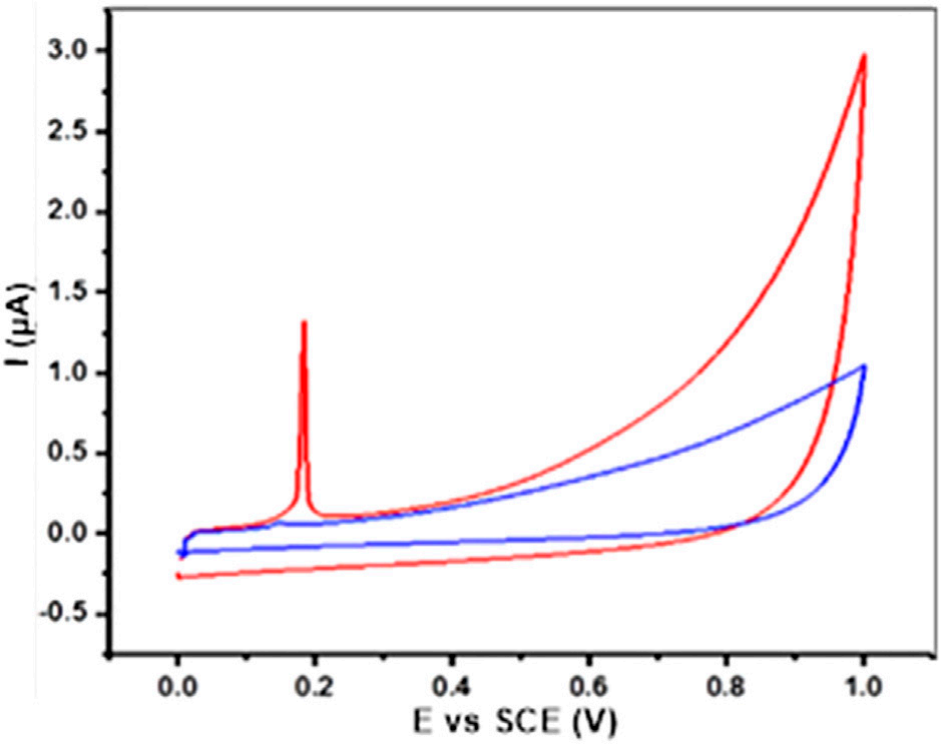
CVs obtained from the AgNP-modified GCE (red line) and bare GCE (blue line) in 0.1 M KCl solution at 50 mV s^−1^.

The electrochemical behavior of AgNPs@OFE was investigated by cyclic voltammetry (CV) at an ensemble electrode. The AgNP ensemble was prepared by the drop-casting method using a glassy carbon electrode (GCE). Figure 7 shows the CV measurement of AgNPs@OFE/GCE, which was recorded in 0.1 M KCl in the potential range of 0–1 V vs. SCE at a scan rate of 50 mV s^-1^. The CV of the bare GCE (blue line) shows no distinctive peaks and only non-Faradaic current, mainly of double-layer charging. On the other hand, the CV at the AgNP-modified GCE (red line) shows the appearance of an oxidation peak at 0.19 V, which is the characteristic of Ag oxidation to Ag^+^ (aq) (Toh et al., 2015; Abbas et al., 2020). This result reveals the electrochemical activity of the synthesized AgNPs@OFE and further confirms their successful synthesis.

### 3.3 Evaluation of the antibacterial activity

**FIGURE 8:**
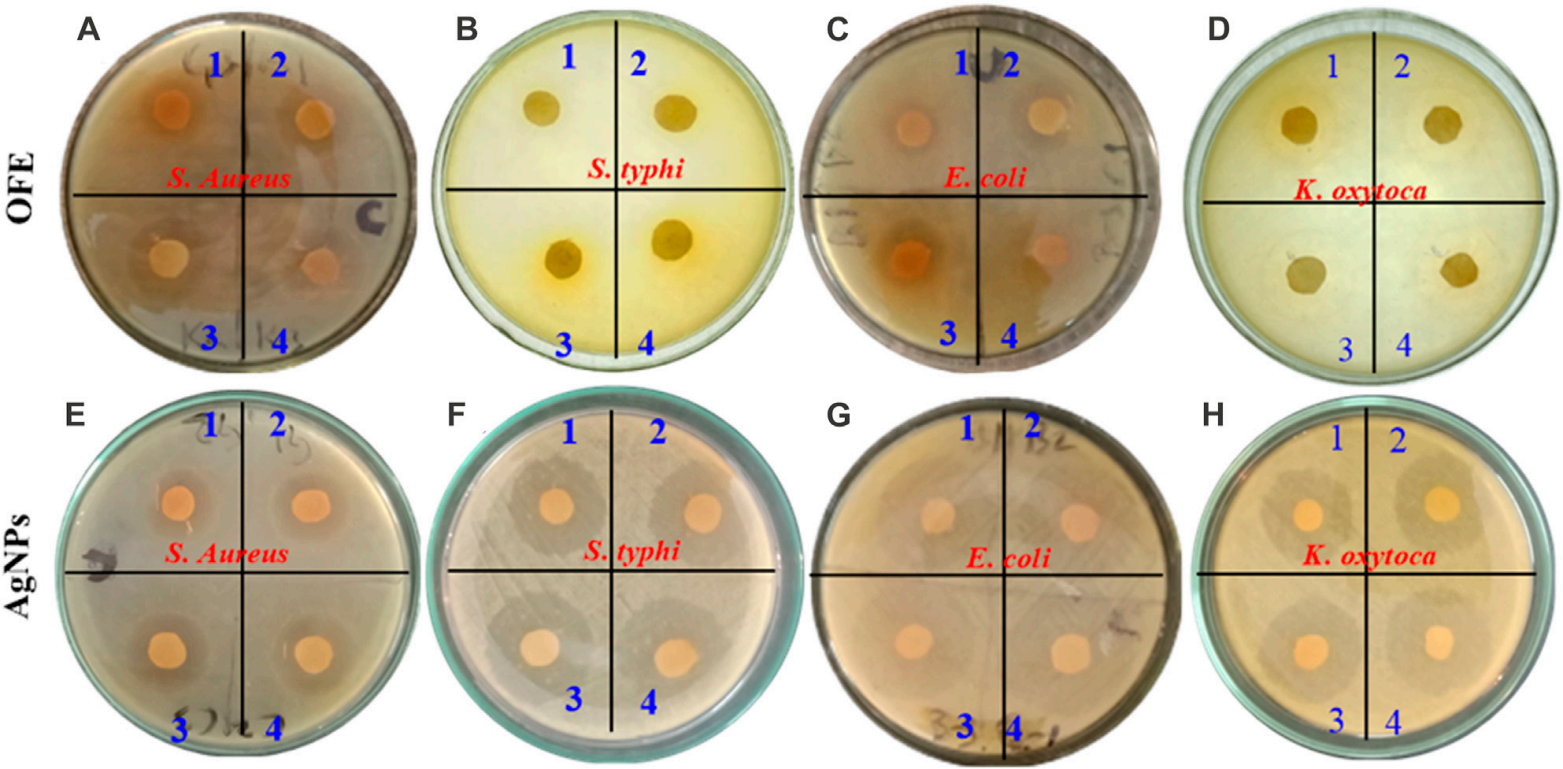
Inhibition zones produced by olive fruit extract **(A–D)** and OFE-capped AgNPs **(E–H)** against four species of pathogens: **(A, E)** *S. aureus*, **(B, F)** *S. typhi*, **(C, G)** *Escherichia coli*, and **(D, H)** *K. oxytoca*. The numbers 1 to 4 refer to increased AgNP concentrations in ascending order.

**FIGURE 9:**
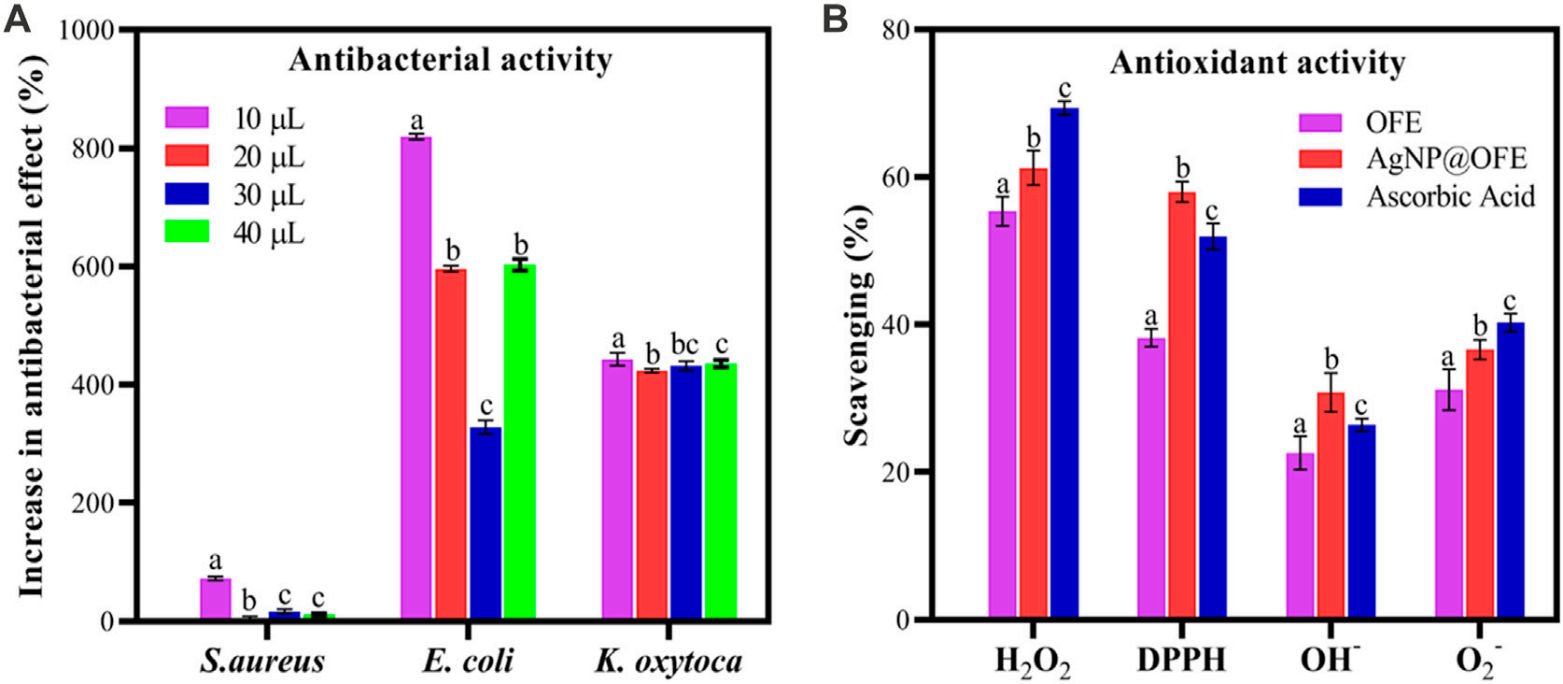
**(A)** Percentage increase in the antibacterial effect of AgNPs@OFE normalized to OFE alone. **(B)** Antioxidant scavenging activity of OFE, AgNPs@OFE, and ascorbic acid against H_2_O_2_, DPPH, HO^−^, and O_2_^−^. Plotted values are the mean of three replicates ± standard deviation. Different alphabets above the different bars in both graphs represent significant difference (*p* < 0.05) in percent increase in the antibacterial effect or antioxidant activity, respectively.

The antibacterial activity of the OFE-mediated AgNPs was determined against three species of Gram-negative bacteria (*E. coli*, *S. typhi*, and *K. oxytoca*) and a Gram-positive bacterium (*S. aureus*). Herein, the semi-quantitative disk diffusion method was chosen with a control experiment with only OFE as a precursor. The zone of inhibition (ZoI) values obtained in the disk diffusion tests are shown in Figures 8A–D for the pure OFE and in Figures 8E–H for the AgNPs@OFE. The values of ZoI are given in Table 1. Interestingly, the pure OFE exhibited no inhibition for *S. typhi*, and only slight inhibition was observed for the other three bacterial strains. In contrast, for AgNPs@OFE, significantly larger inhibition zones were obtained around the disks than those of OFE for the four pathogens. Additionally, the percentage increase in the antibacterial effect of the AgNPs@OFE normalized to the OFE was calculated for the three bacterial strains, as shown in Figure 9A. The data show that the activity improvement by AgNPs@OFE for *S. aureus* was only 10%. In contrast, an antibacterial activity enhancement of approximately 400% and 600% of AgNPs@OFE for *E. coli* and *K. oxytoca*, respectively, compared to the OFE alone, was demonstrated. These results suggest an enhanced antibacterial activity of AgNPs@OFE toward the studied pathogens compared to OFE alone.

**TABLE 1:** Diameter of the inhibition zones (mm) for the tested bacteria using various concentrations of OFE and AgNPs@OFE.

Sample	Volume added from 100 μg/mL	Zone of inhibition (mm)			
	(μL)	*S. aureus*	*S. typhi*	*E. coli*	*K. oxytoca*
OFE	10	3.2 ± 0.1 _a_^a^	ND	2.1 ± 0.1 _a_^a^	3.1 ± 0.1 _a_^a^
	20	5.6 ± 0.1 _a_^b^	ND	3.1 ± 0.1 _a_^b^	3.8 ± 0.1 _a_^b^
	30	6.6 ± 0.1 _a_^c^	ND	5.5 ± 0.2 _a_^c^	4.1 ± 0.2 _a_^c^
	40	7.1 ± 0.1 _a_^d^	ND	3.8 ± 0.1 _a_^d^	4.5 ± 0.1 _a_^d^
AgNPs@OFE	10	5.5 ± 0.2 _b_^a^	16.1 ± 0.2^a^	19.3 ± 0.1 _b_^a^	17.0 ± 0.5 _b_^a^
	20	5.9 ± 0.1 _b_^b^	18.9 ± 0.1 ^b^	21.6 ± 0.2 _b_^b^	19.9 ± 0.7 _b_^b^
	30	7.7 ± 0.1 _b_^c^	20.6 ± 0.2^c^	23.5 ± 0.1 _b_^c^	21.8 ± 0.2 _b_^c^
	40	7.9 ± 0.2 _b_^d^	23.6 ± 0.2 ^d^	26.7 ± 0.2 _b_^d^	24.1 ± 0.4 _b_^d^

Note: the given values are the mean of three replicates ± standard deviation (SD). Different superscripts in the same column for each sample represent the significant difference (*p* < 0.05) among the concentrations, while different subscripts in the same column represent significant difference (*p* < 0.05) between two samples (olive extract and AgNPs).

**SCHEME 2:**
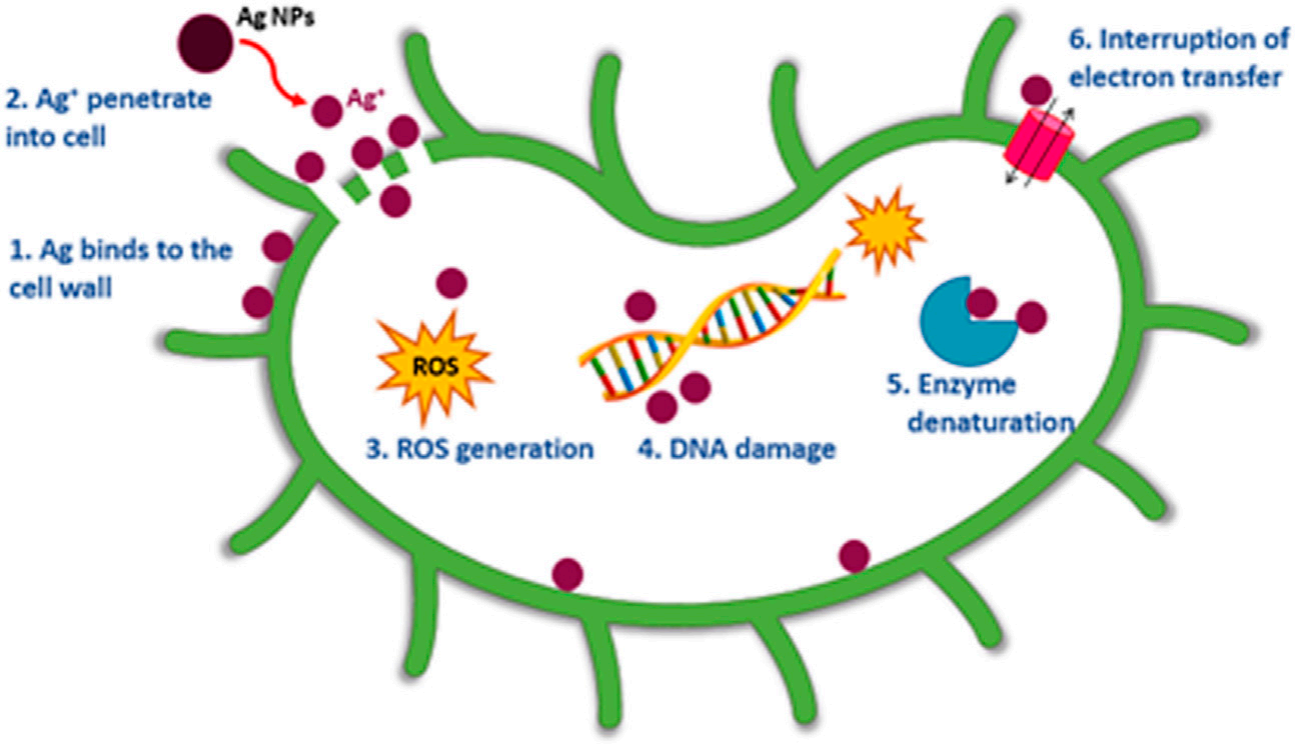
Possible mechanisms of action of AgNPs@OFE as an antimicrobial agent.

Furthermore, AgNPs@OFE exhibited a moderate concentration-dependent antibacterial activity, with an increase in the ZoI as a function of the concentration of AgNPs@OFE (AgNP concentrations in ascending order are denoted on the Petri plates with numbers 1–4), as summarized in Table 1. At smaller concentrations (10 μg mL^-1^) of OFE alone, there was no detectable inhibition for all bacterial strains; thus, the studied concentration range was appropriate for both OFE alone and AgNPs@OFE for comparison purposes. At higher concentrations of AgNPs@OFE, the ZoI would increase, and accordingly, an overlap of the inhibition zones of neighboring disks in the Petri plate will occur, leading to killing all bacteria in the plate. The observed high antibacterial efficiency of AgNPs@OFE is attributed not only to the high concentration of bioactive compounds adsorbed on the surface of the AgNPs@OFE (responsible for capping and stabilizing the particles) but also to the larger surface-to-volume ratio as a characteristic of nanoparticles (Yin et al., 2020). These AgNPs@OFE showed the maximum inhibition against *E. coli* (ZoI of 26.7 ± 0.2 mm) at a concentration of 40 μg mL^-1^, while a minimum inhibition was obtained against *S. aureus* (ZoI = 5.5 ± 0.2 mm) at 10 μg mL^-1^. Additionally, the AgNPs@OFE have shown to be more effective in killing Gram-negative bacterial strains than Gram-negative strains, which is consistent with previous reports on other AgNPs (Mahendran and Kumari, 2016). Furthermore, the antibacterial activity was compared to that of other previously reported functionalized AgNPs. For example, sulfonamide-modified AgNPs showed a ZoI of 29.1, 21.1, and 23.1 mm for *S. aureus*, *S. typhi*, and *E. coli*, respectively, which are comparable to the present study (Amin et al., 2022). The antibacterial activity of our AgNPs@OFE outperformed some previously reported AgNPs such as citrate-capped AgNPs, where a smaller ZoI (7 mm) for *E. coli* was reported (Khatoon et al., 2017). In general, the antibacterial action of silver nanoparticles (AgNPs) is attributed to several mechanisms which involve the interaction between the nanoparticles and bacterial cells. The exact mechanism may vary depending on the specific characteristics of the nanoparticles and bacterial strains involved. Scheme 2 shows some of the main routes of antibacterial actions proposed based on our antibacterial and reactive radical scavenging results and literature reports (Dakal et al., 2016; Ahmad et al., 2020).

1. Adhesion of AgNPs to the cell membrane: This blocks the outer cell walls and hinders transport activities.
2. Disruption of the cell membrane: AgNPs can release Ag^+^ inside and outside of the bacteria. Ag^+^ can penetrate the cell wall and cell membrane, causing leakage of cellular components and loss of membrane integrity, leading to cell death.
3. Generation of reactive oxygen species (ROS): AgNPs could generate ROS such as superoxide radicals (O_2_^•-^), hydrogen peroxide (H_2_O_2_), and hydroxyl radicals (OH^•^). These ROS can cause oxidative stress in bacterial cells by damaging cellular components such as DNA and proteins.
4. DNA damage: AgNPs can bind to the DNA molecule and interfere with its structure and function, causing genetic mutations and cell death.
5. Enzyme denaturation: AgNPs can bind to the active sites of enzymes, inhibiting their activity and disrupting essential cellular processes.
6. Interruption of electron transfer: AgNPs can undergo redox reactions with cellular components, which can disrupt cellular redox balance and interfere with essential metabolic pathways.

### 3.4 Evaluation of antioxidant activities

Various antioxidant assays have been examined in this study because each of the investigated free radicals can exist under different conditions and each assay has its own underlying chemistry, strengths, and limitations. Thus, the versatility of antioxidant assays is useful to obtain a general trend. In all assays, ascorbic acid was used as a standard with the same concentration as the extract (50 μg mL^-1^).

#### 3.4.1 Hydrogen peroxide scavenging activity

The antioxidant scavenging activities (in %) of OFE and AgNPs@OFE for the investigated radicals are compared to the standard antioxidant ascorbic acid as shown in Figure 9B. The hydrogen peroxide scavenging activities (%) of AgNPs, OFE, and ascorbic acid are 55.4 ± 1.9, 61.3 ± 2.3, and 69.4 ± 0.9, respectively, as shown in Figure 9B. Although OFE alone showed the highest hydrogen peroxide scavenging activity, AgNPs@OFE demonstrated a significant hydrogen peroxide scavenging activity close to that of standard ascorbic acid.

#### 3.4.2 DPPH activity

AgNPs, OFE, and ascorbic acid demonstrated scavenging percentages of 48.2% ± 1.2%, 58.0% ± 1.4%, and 52.0% ± 1.8%, respectively, toward the DPPH radical. These results confirm that OFE, AgNPs, and ascorbic acid have comparable scavenging activity toward the DPPH radical.

#### 3.4.3 Hydroxyl radical scavenging activity

The results demonstrated that AgNPs@OFE, OFE, and ascorbic acid showed a scavenging efficiency of 22.0% ± 1.1%, 30.1% ± 1.3%, and 31.5% ± 0.5% for hydroxyl radicals, respectively. OFE and ascorbic acid have similar hydrogen peroxide scavenging potential, while AgNPs@OFE exhibited a lower value but still show a significant antioxidant activity.

#### 3.4.4 Superoxide radical scavenging activity

AgNPs, OFE, and ascorbic acid showed a scavenging efficiency of 31.2% ± 2.8%, 36.6% ± 1.3%, and 40.3% ± 1.2%, respectively, for superoxide radicals.

The antioxidant activity study revealed the highest scavenging activity of AgNPs against H_2_O_2_ (55.4%), followed by DPPH radical (48.2%), O_2_^−^ (31.2%), and OH^−^ (22.0%) radicals. Hence, AgNPs@OFE have high antioxidant activity which is comparable to that of OFE and ascorbic acid. Based on the antibacterial and antioxidant capacity results, we hypothesize that the enhanced activity of AgNPs@OFE is related to a sort of synergistic effect between OFE and AgNP cores in the capped particles. However, each of the two components plays a dominant role in different effects. The antibacterial performance data revealed that the antibacterial effect of AgNPs@OFE mainly originated from the Ag particles, which are known for their high antibacterial activity. On the other hand, the high antioxidant activity was predominated by the presence of OFE as OFE alone showed activity comparable to AgNPs@OFE. This antioxidant activity could be attributed to the phenolic and flavonoid compounds in the OFE that modifies the silver particle surface. Thus, this study provides an insight into the use of olive fruit extract as a potential source of naturally occurring antioxidant. Therefore, the OFE-mediated AgNPs could act as a hydrogen donor, oxygen radical quencher, and reducing agent in further applications.

## 4 Conclusion

Olive fruit extract-mediated biosynthesis of AgNPs (AgNPs@OFE) via sunlight irradiation was reported. The successful synthesis of the particles was characterized by UV-vis, FTIR, and EDX analyses. The OFE comprised bioactive molecules, mainly carboxylated species that are responsible for reducing silver ions to metallic Ag and also act as a capping agent of the produced AgNP. The as-synthesized AgNPs@OFE exhibited uniform spherical particles with a narrow size distribution and an average particle size of approximately 77 nm, as evidenced from SEM imaging. These particles showed high colloidal stability, thanks to the efficient capping effect of OFE. The AgNPs@OFE demonstrated improved antibacterial activity against four different pathogens compared to OFE alone, with the *E. coli* strain exhibiting the highest activity. Furthermore, because of the adsorption of bioactive compounds of OFE, the AgNPs showed comparable antioxidant scavenging activity to the standard antioxidant ascorbic acid against DPPH, hydrogen peroxide, hydroxyl, and superoxide radicals. The enhanced antibacterial and antioxidant capacities of the synthesized AgNPs@OFE were hypothesized to originate from a synergistic effect between core AgNPs and their capping agent OFE. The high stability and considerable antibacterial and antioxidant activities of olive fruit extract-functionalized AgNPs render this simple, eco-friendly approach attractive for upscaling and application in biomedical and nutraceutical developments.

## Data Availability

The raw data supporting the conclusion of this article will be made available by the authors, without undue reservation.
