# Supplementary material for: Biosynthesis of phyto-functionalized silver nanoparticles using olive fruit extract and evaluation of their antibacterial and antioxidant properties
Source: Front Chem. 2023 May 30;11:1202252. doi: 10.3389/fchem.2023.1202252 (PMC10262211; doi:10.3389/fchem.2023.1202252)
Supplement: Supplementary file 1 [file DataSheet1.PDF]

Supporting Information

Biosynthesis of Phyto-Functionalized Silver Nanoparticles Using Olive Fruit Extract and Evaluating their Antibacterial and Antioxidant Properties

Sami Ullah[1#], Rimsha Khalid[1#], Muhammad F. ur Rehman[1], Muhammad I. Irfan[1], Azhar Abbas[1]*, Ali Alhoshani[2], Farooq Anwar[1,3]*, Hatem M.A. Amin[4, 5]*

[1]Institute of Chemistry, University of Sargodha, Sargodha, Pakistan

[2]Department of Pharmacology & Toxicology, College of Pharmacy, King Saud University, Riyadh, Saudi Arabia

[3]Department of Food Science, Faculty of Food Science and Technology, Universiti Putra Malaysia, Serdang, Malaysia

[4]Chemistry Department, Faculty of Science, Cairo University, Giza, Egypt

[5]Analytical Chemistry II, Faculty of Chemistry and Biochemistry, Ruhr University Bochum, Bochum, Germany

[#] These authors contributed equally to this work

*** Correspondence:**
Hatem M.A. Amin
hatem@pc.uni-bonn.de

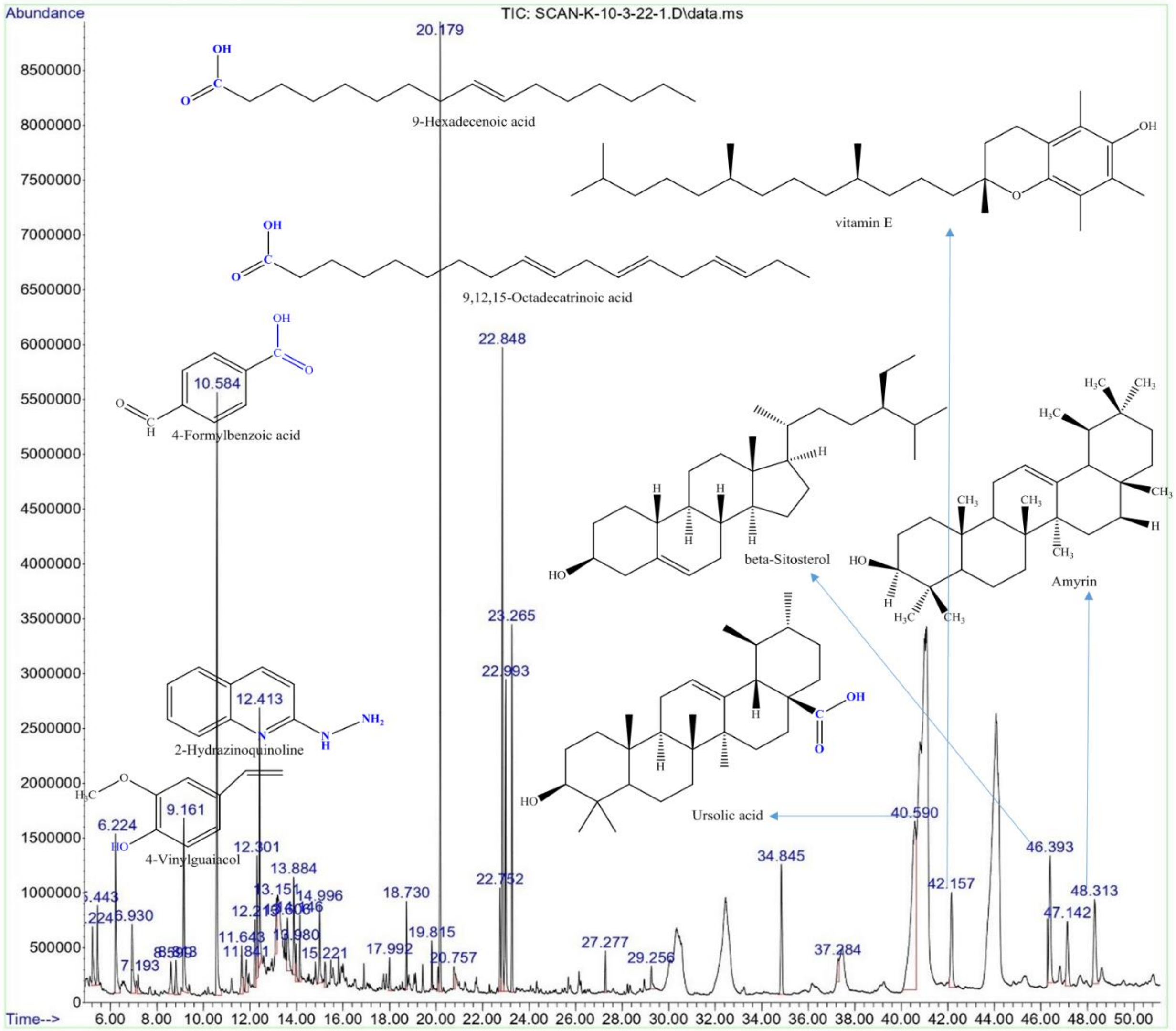

Figure S1. GC-MS chromatogram of olive fruit extract with structure of major detected compounds.

Table S1. Major bioactive compounds identified in the olive fruit extract with their retention time (R_t) and percentage peak area.

**No.**	**R_t (min)**	**Compound Name**	**Peak Area (%)**
1	5.224	Benzoic acid	1.42
2	6.224	Phenol	2.51
3	6.930	2,3-dihydrobenzofuran	1.81
4	7.193	2,3-Anhydro-d-mannosan	0.52
5	8.599	p-hydroxyphenol (Hydroquinone)	0.75
6	9.161	*p*-Vinylguaiacol	3.00
7	10.584	Benzoic acid 4-formyl	9.14
8	11.643	2,3-Difluoroaniline	0.92
9	11.841	Tyrosol	0.94
10	11.841	*trans* -Isoeugenol	0.85
11	12.413	2-Hydrazinoquinoline	3.34
12	13.151	3,4-Altrosan	1.07
13	13.606	Homovanillyl alcohol	1.37
14	13.884	Sinapic acid	1.67
15	13.980	3-fluorobenzoic acid ethyl ester	0.53
16	14.146	4-Ethylsyringol	0.79
17	14.996	Syringlyacetone	1.46
18	15.221	3,4-Dihydroxyphenylglycol	0.53
19	17.992	Orcinol	0.58
20	18.730	Neophytadiene	1.65
21	19.815	Hexadecanoic acid	0.55
22	20.179	9-Hexadecenoic acid	11.47
23	20.757	n-Hexadecanoic acid	0.52
24	22.752	9,12-Octadecadienoic acid	1.34
25	22.848	9,12,15-Octadecatrienoic acid	9.42
26	22.993	Phytol	4.31
27	23.265	Methyl stearate	4.41
28	29.256	Phthalic acid	0.53
29	34.845	Squalene	2.91

30	37.284	Uvaol	0.69
31	40.590	Ursolic	13.88
32	42.157	α- tocopherol	3.74
33	46.409	α -Sitosterol/ β -Sitosterol	3.98
34	47.142	β -Amyrin	2.41
35	48.313	α-Amyrin	3.67
